# Evaluation of Borderline Ovarian Tumor Recurrence Rate after Surgery with or without Fertility-Sparing Approach: Results of a Retrospective Analysis

**DOI:** 10.3390/healthcare11131922

**Published:** 2023-07-03

**Authors:** Basilio Pecorino, Antonio Simone Laganà, Liliana Mereu, Martina Ferrara, Grazia Carrara, Andrea Etrusco, Mariano Catello Di Donna, Vito Chiantera, Giuseppe Cucinella, Fabio Barra, Péter Török, Paolo Scollo

**Affiliations:** 1 Maternal and Child Department, Gynecology and Obstetrics Cannizzaro Hospital, Kore University of Enna, 94100 Enna, Italy; eliopek@gmail.com (B.P.); martiferra@hotmail.it (M.F.); grazia.carrara@studium.unict.it (G.C.); paolo.scollo@unikore.it (P.S.); 2 Unit of Gynecologic Oncology, ARNAS “Civico-Di Cristina-Benfratelli”, 90127 Palermo, Italy; etruscoandrea@gmail.com (A.E.); marianocatellodidonna@gmail.com (M.C.D.D.); vito.chiantera@unipa.it (V.C.); giuseppecucinella@outlook.com (G.C.); 3 Department of Health Promotion, Mother and Child Care, Internal Medicine and Medical Specialties (PROMISE), University of Palermo, 90133 Palermo, Italy; 4 Division of Obstetrics and Gynecology, Department of General Surgery and Medical-Surgical Specialism, University of Catania, 95123 Catania, Italy; liliana.mereu@unict.it; 5 Department of Surgical, Oncological and Oral Sciences (Di.Chir.On.S.), University of Palermo, 90133 Palermo, Italy; 6 Unit of Obstetrics and Gynecology, P.O. “Ospedale del Tigullio”-ASL4, Metropolitan Area of Genoa, 16043 Genoa, Italy; fabio.barra@icloud.com; 7 Department of Health Sciences (DISSAL), University of Genoa, 16132 Genoa, Italy; 8 Department of Obstetrics and Gynecology, Faculty of Medicine, University of Debrecen, 4032 Debrecen, Hungary; petertorokdr@gmail.com

**Keywords:** borderline ovarian tumors, conservative surgery, micropapillary patterns, surgical staging, fertility-sparing surgery

## Abstract

Borderline ovarian tumors (BOTs) comprise 15–20% of primary ovarian neoplasms and represent an independent disease entity among epithelial ovarian cancers. The present study (Clinical Trial ID: NCT05791838) aimed to report a retrospective analysis of the management and outcomes of 86 consecutive BOTs patients, 54 of which were at a reproductive age. All patients with BOTs undergoing surgical treatment from January 2010 to December 2017 were included. Data were retrospectively reviewed. High levels of Ca-125 were observed in 25.6% of the FIGO stage I patients and 58.3% of the advanced disease patients. Fertility-sparing surgery and comprehensive surgical staging were performed in 36.7% and 49.3% of the patients, respectively. Laparotomy was the most frequent surgical approach (65.1%). The most common diagnosis at frozen sections was serous BOT (50.6%). Serous BOTs have significantly smaller tumor diameters than mucinous BOTs (*p* < 0.0001). The mean postoperative follow-up was 29.8 months (range 6–87 months). Three patients experienced a recurrence, with an overall recurrence rate of 3.5% (10% considering only the patients who underwent fertility-sparing treatment). BOTs have low recurrence rates, with excellent prognosis. Surgery with proper staging is the main treatment. Conservative surgery is a valid option for women with reproductive potential.

## 1. Introduction

Borderline Ovarian Tumors (BOTs) are epithelial tumors of the ovaries characterized by up-regulated cellular proliferation and nuclear abnormalities. Nevertheless, in contrast to ovarian cancer, they usually do not show massive stromal invasion [1,2].

Since 1971, classification by the Federation Internationale de Gynecologie et d’Obstetrique (FIGO) recognized BOTs as “low malignant potential” tumors as a distinct entity from ovarian carcinomas. Although they were surgically managed as malignant epithelial ovarian tumors [3,4], the 2014 World Health Organization (WHO) classification describes a BOT as an “atypical proliferative tumor” [5].

BOTs account for 15–20% of all ovarian epithelial neoplasms [1,6]. Six different histological subtypes can be distinguished: serous (50–55%), mucinous (35–45%), endometrioid (2–3%), clear cells (<1%), seromucinous (5–7%), and Brenner tumors (3–5%) [5,7]. Most tumors are diagnosed in young women and as an early-stage disease (FIGO stage I) [8,9,10]. BOTs are characterized by a significantly better prognosis than invasive forms (10-year overall survival rate of 97%) [8,11]. Prognostic factors influencing relapse rates are the advanced-stage of the disease, invasive tumor implants that become similar to low-grade serous carcinoma in the case of serous histology, fertility-sparing procedures such as cystectomy or unilateral salpingo-oophorectomy, intraoperative spillage of the tumor, incomplete surgical staging, and micro-invasive or micropapillary histology [7,12,13]. The standard surgical approach for BOTs is the same as malignant ovarian tumors, except for the need for lymphadenectomy during the surgical staging [6,14,15]. In the case of young women with an early-stage tumor (FIGO stage I–II), conservative surgical treatment is suitable to preserve fertility associated with a close follow-up [9,16,17]. To date, there is no convincing evidence of the efficacy of adjuvant chemotherapy and/or radiotherapy in improving the prognosis [3,11]. Considering these elements, the aim of this retrospective analysis was to review evaluating histology, serological characteristics, therapy, and recurrence rate after surgery with or without a fertility-sparing approach in a large consecutive series of BOTs.

## 2. Materials and Methods

### 2.1. Study Design and Data Collection

From January 2010 to December 2017, all consecutive patients from a single center (Cannizzaro Hospital, Catania, Italy) undergoing surgical treatment for BOTs were included in the current analysis (Clinical Trial ID: NCT05791838). The Institutional Review Board of the Cannizzaro Hospital (approval ID: 27/2022) approved the design, analysis, interpretation of data, drafting, and revisions. The study conformed with the Helsinki Declaration, the Committee on Publication Ethics guidelines, and the Strengthening the Reporting of Observational Studies in Epidemiology (STROBE) Statement [18], validated by the Enhancing the Quality and Transparency of Health Research Network. The data collected were anonymized, considering the observational nature of the study, and did not include personal data that could lead to formal identification of the patient. This study was not publicized. Patients did not receive any remuneration to give consent to be enrolled in this study. Each patient signed an informed consent form to allow data collection for research purposes.

Inclusion criteria were age > 18 years, patients affected by BOT of any histological type and any FIGO stage, and women undergoing surgical treatment with both laparoscopic and laparotomic approaches. Clinical and demographic patient characteristics, including age; BMI; preoperative Cancer Antigen-125 (Ca-125), Carcinoembryonic Antigen (CEA), Cancer Antigen-19.9 (Ca-19.9), and Cancer Antigen-15.3 (Ca-15.3) levels; clinical stage according to the FIGO classification; histopathologic subtype; tumor diameter; tumor implants; and results of frozen sections analysis were recorded. Moreover, the surgical approach and the type of surgical staging were reviewed. Finally, the type of adjuvant treatment, data regarding recurrence of the disease, follow-up after surgery, and treatment for the first relapse were evaluated.

In order to compare our findings with the previous published data, we performed a literature search on PubMed, selecting relevant studies based on the number of enrolled women, consistency of data reporting and adequate follow-up length.

### 2.2. Surgical Treatment

The laparotomic vs. laparoscopic approach was chosen based on the tumor size and the risk of intraoperative cyst rupture. In the case of laparoscopic surgery, an endobag was used to remove the ovarian lesion, avoiding tumor spillage. Peri- and post-menopausal patients who completed their fertility underwent a complete surgical treatment that included both comprehensive staging and the removal of all macroscopic tumor lesions within the abdomen. Complete staging included hysterectomy, bilateral salpingo-oophorectomy, omentectomy (infracolic or total), peritoneal washing with cytology, resection of peritoneal lesions, and systematic peritoneal biopsies in all areas of the abdomen. Pelvic and paraaortic lymphadenectomy or sampling were performed in the case of bulky nodes or advanced FIGO stage disease. Appendectomy was performed in mucinous BOTs to exclude the possibility of ovarian metastasis of mucinous tumors of the appendix. Patients with childbearing potential underwent fertility-preserving surgery, such as unilateral salpingo-oophorectomy, unilateral salpingo-oophorectomy with contralateral ovarian biopsy, unilateral salpingo-oophorectomy with contralateral cystectomy, monolateral cystectomy, bilateral cystectomies, or monolateral cystectomy with contralateral ovarian biopsy and bilateral ovarian biopsy.

### 2.3. Statistical Analysis

Descriptive statistics were performed for characteristics of patients. The Chi-square, Fisher exact and Mann–Whitney U tests were used when comparing categories against categorical and continuous data, respectively. A *p* value < 0.05 was considered statistically significant. SPSS software (SPSS version 21.0, SPSS Inc, Chicago, IL, USA) was used for all statistical evaluations.

## 3. Results

A total of 86 consecutive BOT patients undergoing surgical treatment during the study period were included and analyzed. The median age at diagnosis was 46.1 years (range 17–88). A total of 29 (33%) patients were <40 years old. At the time of diagnosis, 54 patients (62.8%) had childbearing potential, while 29 were in menopause. Seventeen patients underwent previous gynecological surgery: one patient underwent hysterectomy for uterine fibromatosis, two patients underwent hysterectomy with unilateral salpingo-oophorectomy for uterine fibromatosis and non-specified ovarian pathology, and fourteen patients had previously received salpingo-ophorectomy for benign disease. Patient characteristics are shown in Table 1. All the patients underwent an ultrasound scan, and a BOT was suspected according to the International Ovarian Tumor Analysis (IOTA) simple rules. In addition, intraoperative frozen section analysis was conducted in 89.5% (77/86) of the cases; in the other cases, the diagnosis was made upon final histology (without frozen section). Out of eighty-six patients, six patients (7%) had undergone previous surgery at other hospitals with a final diagnosis of BOT. Afterward, these six patients had a comprehensive surgical re-staging.

### 3.1. Clinical Presentation and Tumor Markers

Out of 86, 57 (66.3%) women had symptoms at the time of diagnosis. Thirty patients (52.6%) reported abdominal-pelvic pain, twenty had abdominal swelling, and seven (12.3%) reported menstrual cycle irregularities (spotting, dysmenorrhea, metrorrhagia). Twenty-nine patients with a BOT presented as asymptomatic; their adnexal masses were discovered incidentally during a routine ultrasound examination. As detailed in Table 1, preoperative Ca-125, CEA, Ca-19.9, and Ca-15.3 values were evaluated in all patients. The mean values of tumor markers were Ca-125, 127.88 UI/mL (range 4–2198 UI/mL); CEA, 8.9 ng/mL (range 0.2–495 ng/mL); Ca-19.9, 27.7 UI/mL (range <2.5–279 UI/mL); and Ca-15.3, 23.9 UI/mL (range 5.7–83 UI/mL). Elevated Ca-125 levels were observed in 25.6% of those in stage I. Among the eight patients with elevated values of CEA, seven had a mucinous BOT.

### 3.2. Features of Borderline Ovarian Tumors

#### 3.2.1. Tumor Diameter and Laterality

The mean tumor diameter was 13.8 cm (range 2–45 cm), 25.3 cm (range 5–40 cm), 17.6 cm (range 2–30 cm) and 14.5 cm (range 13–15 cm) for serous, mucinous, serous-mucinous and endometrioid BOTs, respectively. Mucinous BOTs were more likely to have a larger tumor diameter compared to serous BOTs (*p* < 0.0001). In 47.7% of cases (*n* = 41), BOTs involved the right annex; in 30.2% (*n* = 26), the left; and in 22.1% (*n* = 19), both annexes were involved. Fifty-five patients were classified as FIGO stage IA (64%), while seven (8.1%) were stage IB, and twelve (14%) stage IC.

#### 3.2.2. Cases with Co-Incidental Carcinoma

Five patients had a diagnosis of simultaneous cancer (three cases of cervical adenocarcinoma in situ, one patient with endometroid endometrial cancer, and one case with clear cell renal carcinoma).

#### 3.2.3. Micro-Invasive or Micro-Papillary Subtypes

Since the 2014 WHO classification considers the micropapillary variant as a distinct subtype of borderline serous tumor, we analyzed our database and found seven cases (8.1%): five with unilateral disease and two with bilateral BOTs (Table 2). There were nine patients with a BOT and concomitant areas of intraepithelial carcinoma: three cases of serous BOT, five of mucinous BOT, and one with endometroid BOT. Moreover, among these nine patients, two also had microinvasion foci (one serous BOT and one mucinous BOT). Seven patients had positive peritoneal cytology, four patients had epithelial cells referring to BOT, and three had positive malignant cells (one patient with bilateral serous BOT, one with monolateral mucinous BOT, and one with bilateral micropapillary serous BOT). Thirteen patients (15.1%) had non-invasive peritoneal implants. The implants were mainly located in the Douglas pouch, on uterine or tubal serosa, bladder serosa, pelvic peritoneum, rectum epiploic appendix, omentum, or small intestine mesentery. Two patients had invasive peritoneal implants at the level of the pelvic parietal peritoneum: one patient with bilateral serous BOT, and one with recurrence of microinvasive serous borderline tumor. Systematic pelvic lymph node dissections (PLND) were performed in twelve patients (13.9%); among these twelve patients, one underwent PLND during the surgical restaging. The decision to perform PLND was done in the following cases: BOT suspicious for invasion on frozen sections (two patients), presence of bulky lymph nodes (seven patients), concurrent malignant endometrial pathology (one patient with G2 endometrioid adenocarcinoma), and invasive peritoneal implants (two patients).

### 3.3. Surgical Treatment and Staging

Overall, 43 hysterectomies, 73 peritoneal washing with cytology, 16 appendectomies, 41 total omentectomy, and 12 lymphadenectomies or lymph node sampling were performed. A total of 19 patients underwent an omental biopsy, and 59 underwent multiple peritoneal biopsies. Among the 30 mucinous BOT cases, 14 had previously received a salpingo-ophorectomy for benign disease. In our study, laparotomy was the most frequent surgical approach (56 patients, 65.1%). Twenty-eight patients underwent laparoscopic surgery (32.6%). Two conversions occurred, one for the large size of the mass and the other one for the diffuse peritoneal spread of the disease. Six patients received staging surgery due to the previous incidental diagnosis that occurred. The surgical staging of these six patients was carried out either via laparoscopy (*n* = 3) or via laparotomy (*n* = 3). Among them, two underwent unilateral ovarian cystectomy, three unilateral salpingo-oophorectomy, and one patient had a bilateral salpingo-oophorectomy with histological diagnosis resulting in left ovarian serous papillary adenocarcinoma, which was instead classified as borderline serous tumor with focal stromal microinfiltration (FIGO stage IA) at a later revision of the slides.

#### 3.3.1. Radical Surgery

Radical surgery without fertility-sparing intent was performed in 39 patients as total hysterectomy with bilateral salpingo-oophorectomy with or without lymphadenectomy or lymph node sampling, partial or total omentectomy, multiple peritoneal biopsies and appendectomy in the case of mucinous BOTs (Table 3).

#### 3.3.2. Fertility-Sparing Surgery

Fertility-sparing surgery with unilateral salpingo-oophorectomy and/or contralateral ovarian biopsy or mono-bilateral cystectomy was performed in 36.7% of the patients (*n* = 29) (Table 3). Five frozen sections revealed benign pathology, and three cases were incidentally diagnosed with BOT upon histological examination. Restaging surgery was deemed appropriate in half of the cases. In two patients, the second surgical look was negative; residual disease was found in the other two cases, which were both borderline tumors diagnosed incidentally. One patient received a fertility-sparing treatment with peritoneal and omental staging, while the other one received demolitive treatment due to her age and advanced stage of disease. No changes in the FIGO stage were observed in any case. Conservative treatment with close follow-up, diagnostic imaging (CT) and Ca-125 blood dosage were conducted for the remaining four patients.

### 3.4. Intraoperative Frozen Section and Final Histology

Frozen sections were evaluated in 77 patients (89.5%). The most common diagnosis at frozen pathology was serous BOT (*n* = 39; 50.6%), followed by mucinous BOT (*n* = 17; 22.1%), suspected invasive BOT (*n* = 2; 2.6%), and serous-mucinous BOT (*n* = 1; 1.3%). In 18 patients (23.4%), the intraoperative frozen pathology was unable to identify the tumor and reported benign findings. The most common final histology type was serous BOT (*n* = 44; 57.1%). Other histology types were mucinous BOT (*n* = 28; 36.4%), serous-mucinous BOT (*n* = 4; 5.2%), and endometrioid BOT (*n* = 1; 1.3%) on definitive histological examination. The intraoperative rupture of ovarian tumors occurred in three patients (3.5%), two undergoing laparoscopic surgery and one laparotomic surgery. We did not observe any signs of recurrence after 16, 17, and 29 months of follow up, respectively.

### 3.5. Adjuvant and Anti-Estrogen Chemotherapy

Out of eighty-six, only two (2.3%%) patients received adjuvant treatment. In one case, adjuvant chemotherapy (carboplatin plus paclitaxel) was indicated after conservative treatment (laparoscopic right salpingo-oophorectomy plus left cystectomy, Douglas nodulectomy, and infracolic omentectomy) due to the diagnosis of microinvasive bilateral serous borderline ovarian tumor with focal high-grade intraepithelial dysplasia and positive cytology for BOT-compatible cells. The other patient who underwent adjuvant treatment received hormonal therapy (Tamoxifen) after cytoreductive surgery because of advanced disease (multiple peritoneal implants on the external surface of both fallopian tubes, Douglas, and diaphragmatic peritoneum). We did not observe any signs of recurrence after 12 months of follow up.

### 3.6. Follow-Up and Recurrence

The mean postoperative follow-up period was 29.8 months (range 6–87 months). Three (3.5%) patients were diagnosed with recurrence, all of which had an initial fertility-sparing surgery. One of those received adjuvant therapy after primary treatment for microinvasive bilateral serous BOT. The majority of recurrences (2/3) were in the contralateral ovary. All the patients with recurrence underwent a second surgical treatment. Among them, one patient underwent a contralateral salpingo-oophorectomy plus adjuvant chemotherapy as treatment for the first recurrence, and 4 years later received laparoscopic peritoneal biopsies and tamoxifen for the second relapse. The characteristics of the patients with recurrence are shown in Table 4. No patient died from the disease.

Among the potential common elements, all these recurrent cases had stage at least IIB, fertility-sparing surgery unilateral salpingo-oophorectomy, and most of recurrences were in the contralateral ovary.

## 4. Discussion

### 4.1. Fertility-Sparing Surgery in Reproductive Age

This retrospective data analysis reports the main characteristics, either clinical or pathological, of a large consecutive series of BOTs. Based on our results, serous BOTs represent the most common histology, and 62.8% of the patients were with childbearing potential. The conservative treatment is a feasible and safe option, while the overall prognosis of BOTs is good. In our study cohort, the patients’ median age was 46.1 years. Specifically, 33.7% (*n* = 29) of the overall population was aged < 40 years, while 48.8% (*n* = 42) was between 40 and 59 years of age. These findings are in line with previous studies showing that BOTs are more common in young women than malignant tumors, which predominantly occur in older ages [1]. Thus, the preservation of the childbearing potential represents a key point for the counselling of women with BOT, and fertility-sparing treatment should be taken into consideration whenever possible [3]. In this scenario, BOTs are often limited to the ovaries, whereas invasive carcinomas usually spread rapidly in adjacent organs [19]. In our series, 86% of the patients with BOTs were staged as FIGO stage I; specifically, a high rate (64%) of cases were diagnosed at stage FIGO IA. A systematic review of 6362 cases conducted by Du Bois et al. found that approximately 80% of the women with BOT are diagnosed in stage I, whereas higher stages are less common [20]. Considering these epidemiologic data, current international guidelines recommend conservative surgery for patients <40 years old who desire future pregnancies [3,4]. In our series, 29 patients received a fertility-sparing surgical treatment. Among them, eighteen patients at FIGO stage I underwent laparoscopic surgery, nine cases at FIGO stage I had surgery with the laparotomic approach, and two patients (FIGO stage II and III, respectively) underwent laparotomic surgery. The mean age of patients treated with conservative surgery was lower than in patients treated with radical surgery: 33 (range 17–46) and 56 years (range 38–88), respectively.

For stage I, in young women, salpingo-oophorectomy or cystectomy could be considered a feasible option if patients agree to close follow up [1]. In our study, eight cystectomies, twelve unilateral salpingo-oophorectomies (USO), nine USO combined with contralateral ovarian procedures, and three bilateral salpingo-oophorectomies with uterus preservation were performed. Among the patients who underwent USO, one patient at FIGO stage IIB developed disease recurrence in the contralateral ovary 22 months after surgery; this patient underwent a second surgery with partial resection of the residual ovary after adequate counseling, aiming to preserve fertility. Subsequent follow-up was negative, and the patient had a natural pregnancy with a regular course.

### 4.2. Predictors of Recurrence

Despite reproductive outcomes being satisfactory after conservative treatment, accumulating evidence has highlighted lower relapse rates in patients who have undergone radical treatment compared with fertility-sparing surgery [14,21]. In our series, all three patients who experienced a recurrence were initially treated with a fertility-sparing strategy. Nevertheless, there is no robust and definitive evidence for patients who have only received ovarian conservative treatment. A recent multicenter study that included 175 patients who underwent conservative management for BOT, with recurrence in 35 of them (20%), sought to identify factors predictive of recurrence [21]. The authors concluded that the recurrence rate in the multivariate analysis was not significantly affected by the type of conservative surgery. These findings are in line with a previous large observational study of 535 BOT patients undergoing conservative treatment conducted by Delle Marchette et al. [22]. The authors did not show any association between the type of conservative surgery (salpingo-oophorectomy vs. cystectomy) and the risk of recurrence (HR = 1.34; 95% CI 0.98–1.81; *p* = 0.06). Furthermore, a recent multicenter study by Capozzi et al. reported that ultrasound features of the ovarian lesions, such as and cysts with >4 papillae, multilocular cysts > 10 loculi, and maximum diameter > 50 mm, were independent predictive factors of BOT recurrence [23]. Indeed, to date, there are no clear criteria for the selection of patients with BOTs undergoing conservative management who are at high risk of recurrence, and a close follow up is required. However, there is no clear consensus on the standard of the type and frequency of follow up for BOT patients after primary treatment [11]. In this series, patients were evaluated every 3 months for the first year after surgery, then every 6 months during the second year, and gradually at increasing intervals. During follow up, patients underwent a control visit, ultrasound, and serological tumor markers evaluation. In the case of suspicion of recurrence, they were referred to further diagnostic investigations, such as an abdominal CT scan or MRI. However, other groups suggest that women should be given a follow up every 6 months or annually [4]. In our study, the mean follow-up period was 29.8 months (range: 0–87 months). During the follow up, one patient (1.2%) died from causes not related to BOTs, and only 3 out of 86 patients had a relapse, accounting for a recurrence rate of 3.5%. Furthermore, these three patients belong to the group undergoing fertility-sparing surgery (*n* = 3/29; 10%) and developed disease recurrence at 22, 47, and 48 months, respectively. The 10% recurrence rate among patients conservatively treated, based on our results, is slightly lower than in the literature. Overall, recurrence rates are estimated between 11.5% and 13.9% [10,17]. During follow up, ultrasound may represent the best method to detect a lesion in the residual ovary in case of BOTs at an early stage. However, in the case of a serous BOT stage II-IV tumor, the greatest risk is the transformation into low-grade invasive serous carcinoma, which occurs more frequently with peritoneal carcinomatosis [4]. Histopathological characteristics that could play a role in the risk of recurrence are the presence of a microinvasive or micropapillary architecture. The term “microinvasion” has been used in the 2014 WHO classifications for the presence of clusters cells within the stroma, with large eosinophilic cytoplasm similar to epithelial cells coating the surface of papillae [5,7] with a maximum 5 mm extension in the largest linear dimension [7]. In the same WHO classification, the micropapillary variant of Serous Borderline Tumor (SBT) is considered a specific subtype of SBT and represents about 5–15% of SBT [7,24,25]. Initially, this variant was described as “non-invasive low-grade serous carcinoma” (non-invasive LGSC) by Kurman [5,24,25], and this term was adopted as a synonym for “SBT-micropapillary variant” [6]. The absence of hierarchically branched papillae is a characteristic of micropapillary, showing a cribriform epithelial lining of the cyst walls or large fibrovascular papillae or elongated filiform micropapillae (length/width ratio 5:1), with cribriform growth >5 mm in one dimension or at least one area of the continuous micropapillary [7]. Different from the previous WHO classification, novel cytological criteria were indicated for micropapillary SBT diagnosis, which requires the presence of “nuclear atypia greater than that present in cases of conventional SBT”, with a high nucleus-cytoplasm ratio and a small but prominent red nucleolus and rounded cells with a lack of cilia. Kurman defined the micropapillary variant as an intermediate step of progression from the typical serous variant to low-grade serous carcinoma (LGSC) [7]. However, conflicting results were reported in the literature for the prognostic impact of these features [11], and micropapillary patterns and stromal microinvasion have been reported as histological risk factors by some authors [26] but not by others [11,27,28]. Data from a large multicenter retrospective–prospective cohort study with 950 patients reported that neither microinvasion (HR 1.737; 95% CI 0.877–3.439; *p* = 0.1132) nor micropapillary growth pattern (HR 1.688; 95% CI 0.975–2.923; *p* = 0.0618) showed any significant impact on disease recurrence [29]. The micropapillary pattern alone does not seem to have an impact on survival or a reduction of disease-free survival, while it may play a role only if associated with invasive peritoneal implants [7]. In our series, we reported seven patients with BOTs and micropapillary serous variant. The incidence of micropapillary BOTs in our study was 8.1%, in line with literature data. Among these seven patients, two had involvement of both ovaries: one was a FIGO stage IB patient with positive cytology and an absence of peritoneal implants, while the other was a FIGO stage IIIA patient that had both a non-invasive peritoneal implant and a peritoneal lavage positive for borderline-like tumor cells (Table 4). Notably, in our series, two out of three patients with recurrence had a micropapillary pattern of growth. These results, in contrast with some data in the literature, may be explained by the small sample size of our population and the low number of events.

### 4.3. Role of Biomarkers: Differential Diagnosis and Recurrence

The measurement of tumor markers (Ca-125, Ca-19.9) is part of the initial diagnostic process in the case of suspicious BOTs, but only approximately 40% of women have elevated levels of Ca-125. In our series, high Ca-125 values were found in 34.9% of the patients, fully in line with the literature [30]. In the study by Kolwijck et al. [31], mean pre-operative Ca-125 levels were found to be significantly higher for patients with serous and advanced BOTs compared to mucinous and FIGO stage I tumors. However, serum Ca-125 levels can be misleading, and they are not specific for the diagnosis of BOTs. Indeed, false positive results can also be found in women with benign diseases extending across the peritoneal surface, such as endometriosis or in the case of endometriomas or abscesses. The study by Van Calster et al. showed similar serum Ca-125 levels in women with endometriosis, endometriomas, or abscesses, compared to those seen in patients with BOTs [32]. Despite these limitations, the preoperative Ca-125 value’s dosage remains helpful for the diagnosis of indeterminate ovarian mass on imaging, while it may play a beneficial role in the early detection of relapse during the recurrence of BOTs [11].

### 4.4. Strength and Limitations

Several elements should be taken into account for a proper data interpretation: first of all, we described a heterogeneous population of patients with BOTs; in addition, our study is limited by its retrospective design, small sample size, and short duration of follow up. Finally, we lack robust data on the fertility outcome of patients undergoing conservative management. Despite these limitations, the large series of consecutive patients with BOT, the standardized treatment and follow up, and the consistent systematic reporting of clinic-pathological features represents the main strong points of the study, allowing comparative analysis with future research on the same topic.

## 5. Conclusions

Overall, BOTs have a good prognosis with low recurrence rates, even in the cases of conservative treatment. Fundamental points for ideal management include appropriate surgical staging, intraoperative tissue sampling, and appropriate follow up. However, there is a need to identify accurate predictive markers for the risk of relapse. Lastly, the balance between fertility-sparing surgery and risk of recurrence is still a central issue in the management of BOTs; on the one hand, the management of BOT in reproductive aged women should aim for fertility-preservation whenever clinical conditions allow it, whereas more radical surgery can be considered for postmenopausal women; on the other hand, cases with stage ≥ IIB and particular histology, such as micropapillary variants, may be considered for a strict follow-up in order to detect recurrences, even after total hysterectomy and bilateral salpingo-oophorectomy. Further investigations with larger samples and longer follow ups would help to clarify the best strategy to manage patients with BOTs.

## Figures and Tables

**Table 1:** Demographic characteristics and clinical–pathologic data.

Variables	Total
Age years, median (range)	46.1 (17–88)
Age < 40 years, *n* (%)	29 (33.7)
Age 40–49 years, *n* (%)	21 (24.4)
Age 50–59 years, *n* (%)	21 (24.4)
Age > 60 years, *n* (%)	15 (17.5)
Postmenopausal status *n*, (%)	32 (37.2)
Histology	
Serous, *n* (%)	41 (47.7%)
Serous micropapillary, *n* (%)	7 (8.1%)
Mucinous, *n* (%)	30 (34.9%)
Endometrioid, *n* (%)	3 (3.5%)
Serous-mucinous, *n* (%)	5 (5.8%)
Elevated serum markers *	
Ca-125 UI/mL, *n* (%)	30 (34.9)
Ca-19.9 UI/mL, *n* (%)	15 (17.4)
CEA ng/mL, *n* (%)	8 (9.3)
Ca-15.3 UI/mL, *n* (%)	3 (4.6)
Size, median (mm, range)	
Serous	138 (20–450)
Mucinous	253 (50–400)
Endometrioid	176 (20–300)
Serous-mucinous	145 (130–150)
FIGO stage	
IA, *n* (%)	55 (64)
IB, *n* (%)	7 (8.1)
IC1, *n* (%)	9 (10.5)
IC2, *n* (%)	2 (2.3)
IC3, *n* (%)	1 (1.2)
IIA, *n* (%)	0
IIB, *n* (%)	5 (5.8)
IIC, *n* (%)	2 (2.3)
IIIA, *n* (%)	3 (3.5)
IIIB, *n* (%)	2 (2.3)
IIIC, *n* (%)	0
IV, *n* (%)	0
Frozen section result, *n* (%)	77 (89.5)
Benign, *n* (%)	18 (23.4)
Borderline, *n* (%)	57 (74)
Suspicious for invasion, *n* (%)	2 (2.6)
Malignant, *n* (%)	0
Tumor implants	
Non-invasive, *n* (%)	13 (15.1)
Invasive, *n* (%)	2 (2.3)

FIGO: International Federation of Gynecology and Obstetrics. * Range for Ca-125: 4–2198 UI/mL; for CEA: 0.2–495 ng/mL; for Ca-19.9: <2.5–279 UI/mL; for Ca-15.3: 5.7–83 UI/mL.

**Table 2:** Characteristics of patients with microinvasive or micropapillary Borderline Ovarian Tumors.

Age	RO	LO	Treatment	Stage	Fu (Months)	Recurrence
53	Serous micropapillary	/	CS	IA	8	−
57	Serous micropapillary	Serous micropapillary	CS	IB	22	−
41	Serous micropapillary	/	CS	IA	24	−
21	/	Serous micropapillary	USO	IIB	22	+
37	/	Serous micropapillary	USO	IA	22	−
54	Serous micropapillary	Serous micropapillary	CS	IIIA	27	−
51	Serous micropapillary	/	CS	IA	75	−
29		Serous micropapillary	USO and CT	IIC	48	+
37	Serous microinvasive	Serous microinvasive	USO + C and CT	IIC	54	+

RO: right ovary; LO: left ovary; CS: complete cytoreductive surgery; USO: unilateral salpingo-oophorectomy; AT: adjuvant therapy; CT: chemotherapy; C: cystectomy.

**Table 3:** Treatment modalities.

Variables	Total
Surgical treatment	
Radical, *n* (%)	39 (49.4)
Conservative, *n* (%)	29 (36.7)
Fertility-sparing surgical procedures	
Unilateral cystectomy, *n* (%)	5
Bilateral cystectomy, *n* (%)	3
USO, *n* (%)	12
USO and contralateral ovarian Bx, *n* (%)	2
USO and contralateral cystectomy, *n* (%)	7
Bilateral salpingo-oophorectomy without hysterectomy, *n* (%)	3 (3.8)
Surgical approach	
Laparoscopy, *n* (%)	28 (32.6)
Laparotomy, *n* (%)	56 (65.1)
Adjuvant therapy	
Chemotherapy, *n* (%)	1 (1.15)
Tamoxifen, *n* (%)	1 (1.15)

USO: unilateral salpingo-oophorectomy; Bx: biopsy.

**Table 4:** Characteristics of patients with recurrence.

Age	Histology	Stage	Initial Treatment	Recurrence Site	Time to First Recurrence (Months)
21	Serous micropapillary	IIB	USO	Contralateral ovary	22
37	Serous micropapillary bilateral	IIC	USO + unilateral cystectomy	Contralateral ovary	54
29	Serous	IIC	USO + USO	Peritoneum	52

USO: unilateral salpingo-oophorectomy; BSO: bilateral salpingo-oophorectomy.

## Data Availability

A full anonymized dataset will be available from the corresponding authors upon reasonable request.
